# Copper Oxide Nanoparticle-Based Immunosensor for Zearalenone Analysis by Combining Automated Sample Pre-Processing and High-Throughput Terminal Detection

**DOI:** 10.3390/s21196538

**Published:** 2021-09-30

**Authors:** Zhihong Xuan, Yanxiang Wu, Hongmei Liu, Li Li, Jin Ye, Songxue Wang

**Affiliations:** Academy of National Food and Strategic Reserves Administration, No.11 Baiwanzhuang Str, Xicheng District, Beijing 100037, China; xzh@ags.ac.cn (Z.X.); wyx@ags.ac.cn (Y.W.); lhm@ags.ac.cn (H.L.); ll@ags.ac.cn (L.L.)

**Keywords:** zearalenone, high-throughput pre-treatment, CuO NPs, automatic signal conversion

## Abstract

A rapid and high-throughput fluorescence detection method for zearalenone (ZEN) based on a CuO nanoparticle (NP)-assisted signal amplification immunosensor was developed using an automated sample pretreatment and signal conversion system. CuO NPs with high stability and biocompatibility were used as carriers to immobilize anti-ZEN antibodies. The obtained CuO NP-anti-ZEN can maintain the ability to recognize target toxins and act as both a signal source and carrier to achieve signal conversion using automated equipment. In this process, target toxin detection is indirectly transformed to Cu^2+^ detection because of the large number of Cu^2+^ ions released from CuO NPs under acidic conditions. Finally, a simple and high-throughput fluorescence assay based on a fluorescent tripeptide molecule was employed to detect Cu^2+^, using a multifunctional microporous plate detector. A good linear relationship was observed between the fluorescence signal and the logarithm of ZEN concentration in the range of 16.0–1600.0 μg/kg. Additionally, excellent accuracy with a high recovery yield of 99.2–104.9% was obtained, which was concordant with the results obtained from LC-MS/MS of naturally contaminated samples. The CuO NP-based assay is a powerful and efficient screening tool for ZEN detection and can easily be modified to detect other mycotoxins.

## 1. Introduction

Zearalenone (ZEN) is a nonsteroidal estrogenic mycotoxin produced by several species of *Fusarium*, particularly *F. graminearum*, *F. culmorum*, *F. equiseti*, and *F. verticillioides* [1]. ZEN and its derivatives have been widely detected in agricultural products, specifically wheat, corn, barley, soybeans, and oats [2]. ZEN with high thermostability can accumulate in the human body through food chains and cause strong mutagenicity, teratogenicity, neurotoxicity, reproductive toxicity, and carcinogenicity [3,4]. To protect humans from ZEN exposure and reduce economic losses, national and international agencies adopted regulations for the control of ZEN contamination. Thus, the maximum residue level of 350 µg/kg in unprocessed corn was set by the European Union (Commission Regulation, EC No. 1126/2007). China has set a maximum level of 60 µg/kg for ZEN in cereal and cereal-based products (National Food Safety Standard GB2761-2017). Based on the important effects of ZEN on human and animal health, monitoring of ZEN in food and feed is a global priority for protecting customers’ health and facilitating international food and feed trade.

Therefore, extensive research has been conducted to develop sensitive and specific methods for rapid and economic ZEN detection. High-performance liquid chromatography (HPLC) [5] and liquid chromatography combined with mass spectrometry (LC-MS/MS) [6,7] are the most commonly used accurate quantitative methods because of their high selectivity and accuracy. However, these methods are time-consuming; require harmful solvents; and depend on professional technology, costly instruments, and tedious sample pretreatment. Conventional lateral flow immunoassays (LFA) with gold nanoparticles (AuNPs) or fluorescent labels [8,9], and as well as enzyme-linked immunosorbent assays (ELISAs) [10], are simple and convenient; however, these methods suffer from low sensitivity and poor quantitation, limiting their further applications to complex sample matrices. In order to overcome the limitations of traditional analytical methods, numerous studies have focused on more cost-effective and simple methods for ZEN analysis. These methods include magnetic bead (MB)-based methods [11], electrochemistry-based methods [12,13], microchip-based methods [14], quantum dot (QD)-based methods [15], surface-enhanced Raman scattering (SERS)-based assays [16], metal–organic frameworks (MOFs)-based methods [17], and liposomes-based methods [18,19]. Among them, MB-based methods have shown important advantages in detecting mycotoxins [20,21]. MBs have high surface areas for immobilizing large numbers of reagents and contribute to the increase in reaction rate due to the full contact between the capture probe on MB and the target in the reaction mixture [22]. Our previous studies developed a MB-based purification assay to achieve automated and high-throughput sample preprocessing and reduce matrix effects [23,24,25]. Although the constructed method is an effective tool for mycotoxins purification, terminal detection relies on large instruments [23,25] or can only be used to detect individual mycotoxin following purification [24]. Therefore, the goal of this study was to combine automated sample preprocessing with high-throughput terminal detection for efficient and rapid detection of mycotoxins.

The use of copper oxide nanoparticles (CuO NPs) as intermediates is pivotal for the integration of automated sample preprocessing and high-throughput terminal detection to enhance the detectable signal and improve detection efficiency. As nanostructured metal oxides, CuO NPs show brilliant physical and chemical properties due to the quantum confinement in the nano-dimensions, such as good biocompatibility, high specific surface area, antibacterial, low cost, non-toxicity, as well as excellent stability in solution [26,27,28,29]. Lai et al. [30] reported a sensitive detection of carcinoembryonic antigen based on CuO NP-labeled antibody. Li et al. [31] demonstrated a metalloimmunoassay for human immunoglobulin G (IgG) detection through CuO NPs labeling. Liu et al. [32] developed a fluorescence immunoassay for prostate-specific antigen detection with CuO NPs as signal labels; after dissolving CuO NPs with acid, the fluorescence signal of SGHK-Dns can be quenched by Cu^2+^ with high quenching efficiency of 84.8%, which can be used for the sensitive detection of Cu^2+^. Therefore, a CuO NPs-based assay for developing simple and sensitive immunosensors has wide application and the unique advantages of the CuO NPs-based assay still remain to be exploited.

In this study, we developed a CuO NP-based immunosensor for ZEN detection by combining automated sample preprocessing with high-throughput terminal detection. The method was based on a three-component competitive immune reaction achieved by an automatic instrument, through which the target toxin content could automatically be converted into the Cu^2+^ concentration. The Cu^2+^ can be detected with a high-throughput fluorescence method. Under optimized conditions, the CuO NP-based method has excellent accuracy with high recovery, and the test results are concordant with those obtained from LC-MS/MS for real sample analysis. The designed CuO NP-based assay provides a versatile platform for the development of fast and high-throughput mycotoxin pretreatment and terminal detection. 

## 2. Materials and Methods

### 2.1. Reagents and Chemicals

HPLC grade acetonitrile (CAS: 75-05-8) and methanol were obtained from Merck (Darmstadt, Germany). A standard solution of ZEN (1.96 mg/L) (CAS:17924-92-4) was purchased from Biopure (Tulln, Austria) and was stored at −18 °C. Complete antigen (ZEN-BSA) and monoclonal ZEN antibody (anti-ZEN) were acquired from Wuxi Jieshengjiekang Biological Technology Co., Ltd. (Beijing, China). CuO nanoparticles (CuO NPs, CAS: 1317-38-0) were obtained from Sigma-Aldrich (Milwaukee, Brookfield, WI, USA). NHS-activated MB, which can combine antibodies directly without the EDC activation step, was purchased from Shanghai enriching biotechnology Co., Ltd. (Shanghai, China). Morpholinoethanesulfonic acid (MES, CAS: 4432-31-9) and bovine serum albumin (BSA, CAS: 13755-38-9) were acquired from Beijing Solarbio Science & Technology Co., Ltd. (Beijing, China). Tween-20 (CAS: 9005-64-5) was supplied by Beijing Aipoo HuaMei Biotechnology Co., Ltd. (Beijing, China). SGHK-Dns was synthesized and purchased from ChinaPeptides Co., Ltd. (Beijing, China). Blank maize and wheat samples were acquired from China. The ultrapure water was purified in the experiment by a Water Purifier purification machine (Chengdu, China)

### 2.2. Apparatus and Measurements

LC-MS/MS analysis was performed by using Agilent ultra-HPLC system and an Agilent 6470 QQQ-MS (Agilent Technologies). Data acquisition as well as qualitative and quantitative analysis were realized by using Agilent Mass Hunter Workstation software. Fluorescence signals were collected on a Multimode Microplate Reader (PerkinElmer/Victor Nivo 3S) with the excitation and emission wavelengths of 355 nm and 530 nm, respectively. The size distribution and zeta potential were measured and statistics were analyzed by a Zeta-sizer Nano-ZS90 instrument. A Mycotoxin Automatic Purifier (JJHZ10, Beijing Dongfu Jiuheng Instrument Technology Co., Ltd.) with minor change about the software determining the sequence of events shown in Table S1 was used for performing the automated magneto-controlled pretreatment and signal conversion process.

### 2.3. Preparation and Characterization of CuO NP-Anti-ZEN Conjugates

The labeling of anti-ZEN with CuO NPs was performed by previous literature [33] in which the nano-copper oxide powder was ultrasounded into PBS buffer solution, which was followed by the adding of antibodies to the formed colloidal solution of CuO NPs. The labeling process in our developed method was as follows: 5 mg of CuO NPs was weighed into 10 mL of PBS buffer solution and sonicated for 15 min, to which 100 µL of monoclonal ZEN antibody (anti-ZEN, the original concentration is 2 mg/mL) was added to the CuO NPs solution by a stepwise process; then, the mixture was shaken for 1 h at 500 rpm. After being centrifuged for 5 min at 6000 rpm, the suspension was discarded, and the functionalized CuO NPs were redispersed in 5 mL of PBS containing 1% BSA to block the unsaturated sites. The only BSA-coated CuO NPs (CuO NPs-BSA) were prepared in the same way except for the addition of an anti-ZEN antibody. In order to confirm the successful decoration of anti-ZEN on CuO NPs, the size distribution and zeta-potential of CuO NPs before and after modification were measured and statistics were analyzed.

### 2.4. Synthesis and Characterization of the Complex (MB-BSA-ZEN) between Magnetic Bead (MB) and Complete Antigen (BSA-ZEN)

MB-BSA-ZEN synthesis was performed according to our previous report [23,24] except that the antibody was replaced by complete antigen with minor changes: a volume of 2.5 mL of NHS activated MB suspension was placed in a 5 mL centrifuge tube, and the supernatant was discarded with the assistance of an external magnetic field. The remaining MB in the tube was washed thoroughly with absolute ethyl alcohol to remove the residual stored reagents on the surface of MB. Subsequently, 1.5 mg of ZEN-BSA in 50 mM PBS buffer containing 1% BSA was added to the suspension of MB. After two hours of incubation, the supernatant was discarded, and the precipitate was blocked by adding 5.0 mL of PBS with 2% BSA as a blocking reagent. The prepared complex (MB-BSA-ZEN) was washed three times with phosphate buffer solution with 0.1% Tween-20 (PBST) and PBS, respectively and stored at 4 °C. The indirect characterization of the complex was performed according to the interaction between CuO NP-anti-ZEN/CuO-BSA and MB-BSA-ZEN. The formed immune complex (CuO NP-anti-ZEN-MB-BSA-ZEN) was washed and separated via an external magnetic field and then digested by HCl; the obtained Cu^2+^ concentration can be an indicator of the successful synthesis of MB-BSA-ZEN and CuO NP-anti-ZEN.

### 2.5. The Study of Fluorescence Stability of SGHK-Dns

The study of fluorescence stability of SGHK-Dns was performed by selecting 0, 20, 50, 100, 200, 500, and 1000 ng/mL Cu^2+^ to react with SGHK-Dns of the same concentration (2 μM); the fluorescence signal was monitored for 1 h by a multifunctional microplate detector in a 96-well plate every 10 min.

### 2.6. Cu^2+^ Detection Procedure

First, 100 μL of SGHK-Dns (2 μM) solution in PBS buffer was added to 100 μL Cu^2+^ solution obtained by the digestion of CuO NPs in acid solution. The fluorescence quenching reaction occurred immediately after the two solutions are mixed. Finally, the fluorescence signals were collected on a Multimode Microplate Reader with the excitation and emission wavelengths of 355 nm and 530 nm, respectively.

### 2.7. Sample Preparation and Extraction

Real wheat and maize samples were collected from Hanan Province, China. All samples were finely ground and filtered through a 40-mesh sieve. The ZEN-spiked samples for recovery experiment were prepared by adding ZEN stock solution to blank samples to obtain final ZEN concentrations at 30, 60, and 120 ng/mL in triplicate. The sample extraction was prepared as follows: first, 5.0 g of finely ground samples were weighed into a 50 mL centrifuge tube and extracted using 20 mL of extraction solvent (acetonitrile/water, 90:10, *v*/*v*) by shaking 20 min on a vortex shaker. Sequentially, the samples were centrifuged for 5 min at 7000 rpm, and the supernatants were collected for further analysis. 

### 2.8. Linear Relationship and Detection Limit

A sample extract containing ZEN was diluted 10-fold before adding to the mixture of CuO NP-anti-ZEN and MB-BSA-ZEN; then, the resulting three-component reaction mixture was incubated for 30 min under stirring conditions with the automated instrument. Under optimal conditions, the linear relationship was established by plotting the logarithmic value of different ZEN concentrations (16, 32, 80, 160, 320, 800, and 1600 ng/mL) versus the corresponding fluorescence intensity. LOD and LOQ were defined as the concentration of toxin equivalent to three times (for LOD) and ten times (for LOQ) the value of the standard deviation (SD) measured in the absence of toxins.

### 2.9. Specificity of the Assay

The specificity of the CuO NP-based method was studied by choosing a series of structurally similar toxins (AFB_1_, OTA, DON, AFB_2_, AFG_1_, AFG_2_) for comparison. The concentrations of AFB_1_, AFB_2_, AFG_1_, and AFG_2_ were 20 ng/mL, whereas the concentrations of OTA, DON, and ZEN were 20 ng/mL, 1000 ng/mL, and 60 ng/mL, respectively. 

### 2.10. Analytical Application for Actual Samples

To evaluate the reliability of the CuO NP-based assay, 10 naturally contaminated samples were detected by the CuO NP-based assay and LC-MS/MS analysis. Sample extraction and LC-MS/MS determination were performed in accordance with our previous study [34]. The grated sample (5.0 g) was extracted with 20.0 mL of acetonitrile/water (90:10, *v*/*v*) by vigorous stirring for 20 min and centrifuged at 6000 rpm for 5 min. The supernatants were collected for the CuO NP-based assay and LC-MS/MS analysis.

## 3. Results and Discussion

### 3.1. The Principle of the CuO NP-Based Immunosensor for ZEN Detection

**Scheme 1:**
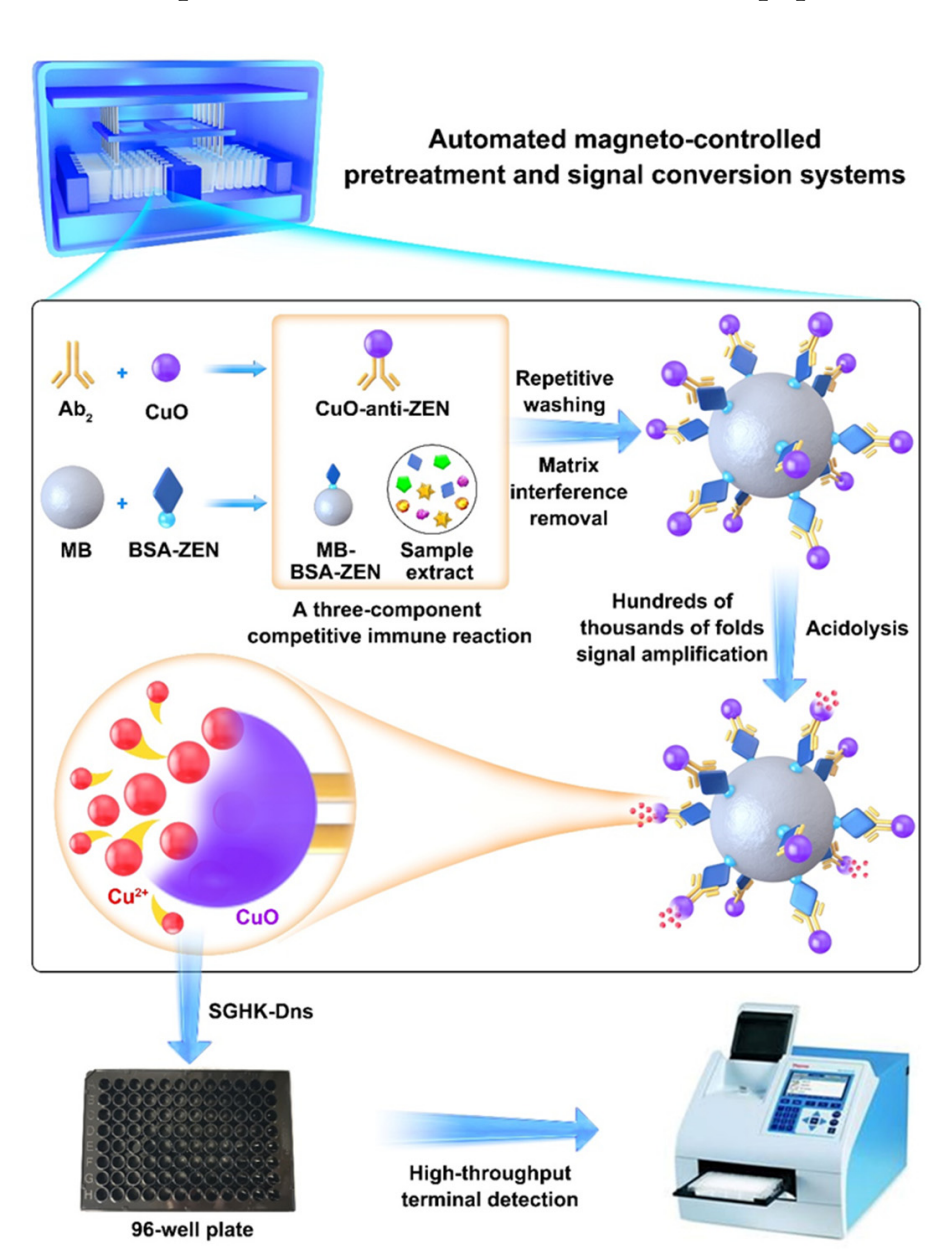
The principle of the automated sample pretreatment and signal conversion system for rapid and high-throughput ZEN detection.

The principle of the CuO NP-based immunosensor for ZEN detection is shown in Scheme 1. First, CuO NPs and magnetic beads (MBs) were modified with antibody (anti-ZEN) and complete antigen (MB-BSA-ZEN) to form CuO NP-anti-ZEN and MB-BSA-ZEN, respectively. Second, by using an automatic sample preprocessing and signal conversion system built in our lab, a three-component competitive immune reaction including target toxins (ZEN), CuO NP-anti-ZEN, and MB-BSA-ZEN was established. Therefore, a larger amount of ZEN in the solution led to fewer CuO NP-anti-ZEN combining with MB-BSA-ZEN. Third, through the automated washing, concentration, and transfer steps, the captured CuO NPs on the surface of MB were treated with an acidic solution (HCl, 10 mM), which could release the corresponding metal ions (Cu^2+^) with an amplification effect of hundreds of thousands of folds. Here, CuO NPs act as the signal source and signal carrier to achieve signal conversion and signal amplification. It is worth mentioning that the amount of released Cu^2+^ or captured CuO NPs was dependent on the target toxin concentration. In this manner, target toxins are indirectly identified via Cu^2+^ detection based on the large amount of Cu^2+^ released from the CuO NPs. Finally, an ATCUN peptide of SGHK labeled with the fluorophore Dns (denoted as SGHK-Dns) was used as the Cu^2+^ sensing probe [32]; thus, Cu^2+^ was easily analyzed in a high-throughput manner using a multifunctional microporous plate detector based on the principle that the fluorescence of SGHK-Dns would be quenched after formation of the Cu^2+^–peptide complex.

### 3.2. Synthesis and Characterization of Antibody (Anti-ZEN)-Labeled CuO NPs

**Figure 1:**
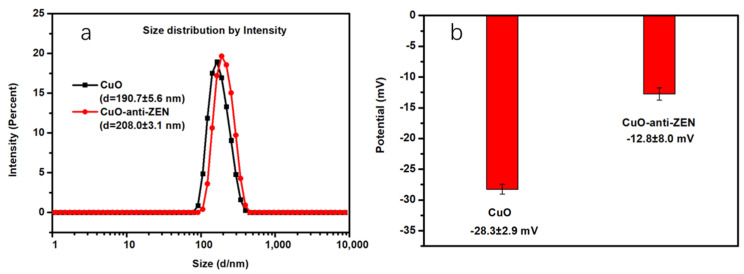
Characterization of antibody-labeled CuO NPs by Zeta-sizer Nano-ZS90 instrument. (**a**) Size distribution and (**b**) zeta potentials of CuO NP colloid and CuO NP-anti-ZEN.

According to previously reported references, the antibody and CuO NPs interact through physical adsorption [35]. Figure 1a shows that CuO NPs had an average hydrated diameter of 190.7 ± 5.6 nm; after the antibody was connected on the surface of CuO NPs, the particle size increased to 208 ± 3.1 nm, which indirectly indicated the successful modification of the antibody (about 17 nm) on CuO NPs. Notably, the size obtained through the dynamic light scattering (DLS) of CuO NPs was much larger than the average size of 40 nm obtained through transmission electron microscopy (TEM). This is because DLS gives a hydrodynamic size corresponding to the core and swollen corona of the micelles, whereas TEM gives the core size for micelles in a dried state, as the corona with low electronic density is not visible. The zeta potential of CuO NPs after conjugating with the anti-ZEN tended to show less negative charge (from −28.3 ± 2.9 to −12.8 ± 8.0 mV, Figure 1b). These data confirm the successful decoration of anti-ZEN on CuO NPs. Studies reported that the changes in the hydrodynamic diameter and zeta potential of CuO NPs before and after modification can reflect the binding of DNA to the CuO NP surface [36]. Thus, the antibody binds to the CuO NP surface in the same manner as DNA.

### 3.3. The Interaction Principle between Cu^2+^ and SGHK-Dns and the Fluorescence Stability Study of SGHK-Dns

A study [32] has shown that Cu^2+^ exhibits both high binding affinity to SGHK peptide and high quenching ability toward the Dns group; after forming the Cu^2+^–peptide complex, the fluorescence signal of SGHK-Dns gradually reduced with increasing Cu^2+^ concentration. Figure S1 showed the structure of SGHK-Dns and the interaction principle between the SGHK-Dns and Cu^2+^. To confirm whether the change in fluorescence intensity of SGHK-Dns is suitable to this experimental system, the fluorescence stability of SGHK-Dns was investigated. Different concentrations of Cu^2+^ (0, 20, 50, 100, 200, 500, and 1000 ng/mL) were reacted with a constant concentration of SGHK-Dns (2 μM). The fluorescence signal was rapidly quenched within a few seconds, and then, the fluorescence intensity remained unchanged within 1 h (Figure S2), demonstrating the stability of the peptide–Cu^2+^ detection system.

### 3.4. Synthesis and Characterization of Complex (MB-BSA-ZEN) between MB and Complete Antigen (BSA-ZEN)

**Figure 2:**
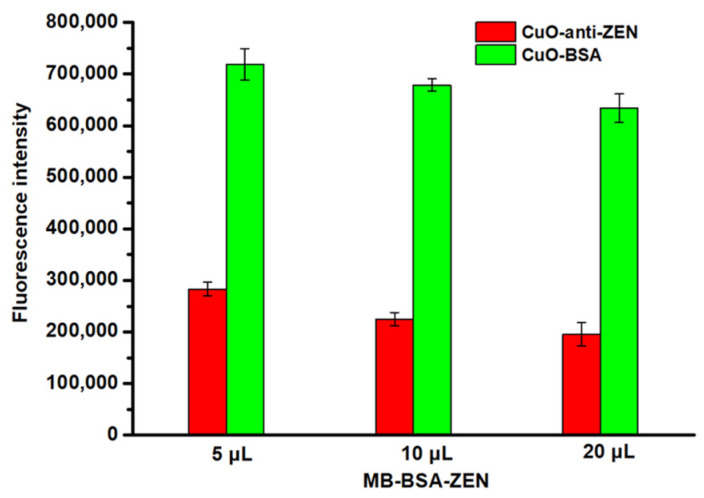
Specific and nonspecific reaction between CuO NP-anti-ZEN/CuO NP-BSA and MB-BSA-ZEN.

We previously characterized the MB and protein complex from the point of view of the maximum saturation supermagnetization; it changes slightly before and after protein modification, and this is enough for magnetic separation under an external magnetic field [23,24,37]. In this study, we used an indirect method to further verify the successful synthesis of MB-BSA-ZEN. Excess CuO NP-anti-ZEN/CuO NP-BSA reacted with different amounts of MB-BSA-ZEN (5, 10, and 20 µL), and the formed immunocomplex was washed and digested with acid solution (HCl, 10 mM). The concentration of the Cu^2+^ obtained was measured through the interaction between Cu^2+^ and SGHK-Dns; higher Cu^2+^ concentration would lead to a lower fluorescence intensity of SGHK-Dns based on the interaction principles between them described in Section 3.3. Thus, as shown in Figure 2, the red bars showing higher Cu^2+^ concentration than the green ones represented the reaction between CuO NP-anti-ZEN and MB-BSA-ZEN, indicating that with increasing amounts of MB-BSA-ZEN, the fluorescence intensity of SGHK-Dns decreased. In addition, the above experimental results indirectly demonstrated the successful synthesis of MB-BSA-ZEN. The green histogram with high fluorescence intensity revealed that there is almost no nonspecific reaction between CuO NP-BSA and MB-BSA-ZEN after washing. These results demonstrate the successful synthesis of CuO NP-anti-ZEN and MB-BSA-ZEN, and the reaction between them is based on specific interactions of the antigen and antibody.

### 3.5. The Study of Nonspecific Adsorption and the Optimal Reaction Time

**Figure 3:**
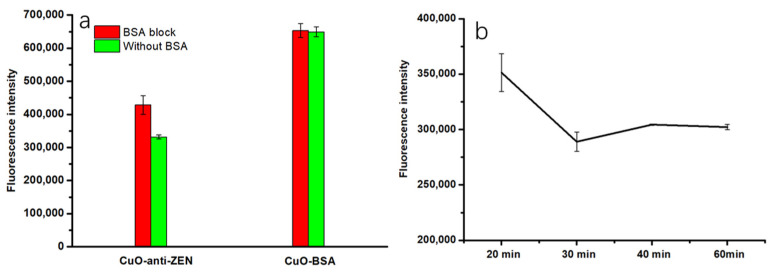
The study of nonspecific adsorption between MB-ZEN-BSA and CuO NP-anti-ZEN with and without BSA (the reaction time is 10 min) (**a**) and the reaction time optimization between MB-ZEN-BSA (5 mg/mL, 10 μL) and CuO NP-anti-ZEN (1 mg/mL, 5 μL) (**b**).

To verify whether nonspecific adsorption between MB-ZEN-BSA and CuO NP-anti-ZEN would occur, bovine serum albumin (BSA), a common blocking reagent, was selected for investigation. As shown in Figure 3a, when CuO NP-anti-ZEN reacted with MB-BSA-ZEN in reaction buffer containing 2% BSA, the obtained fluorescence intensity was higher than that without BSA, which demonstrated that nonspecific adsorption between MB-ZEN-BSA and CuO NP-anti-ZEN occurred, and BSA was able to inhibit nonspecific adsorption. When CuO NP-BSA reacted with MB-BSA-ZEN, fluorescence intensity with BSA was almost the same as that without BSA; additionally, the signal was a little lower than that obtained as the blank test (0 ng/mL) of Figure S2. These results indicate that the reaction between CuO NP-anti-ZEN and MB-BSA-ZEN is attributable to the specific interaction of antibodies and antigens; there is low nonspecific adsorption between MB-ZEN-BSA and CuO NP-BSA.

The optimal reaction time between MB-ZEN-BSA and CuO NP-anti-ZEN was also studied in the developed method. Figure 3b indicates that when the reaction time was 20 min, the fluorescence intensity was almost 350,000, and the fluorescence intensity gradually decreased to about 290,000 when the reaction time was 30 min. Thereafter, the Cu^2+^ signal intensity decreased slightly when the reaction time continued to extend to 1 h. Therefore, in consideration of the sensitivity of the constructed method, the optimal reaction time was set at 30 min for this study.

### 3.6. Analysis Performance

**Figure 4:**
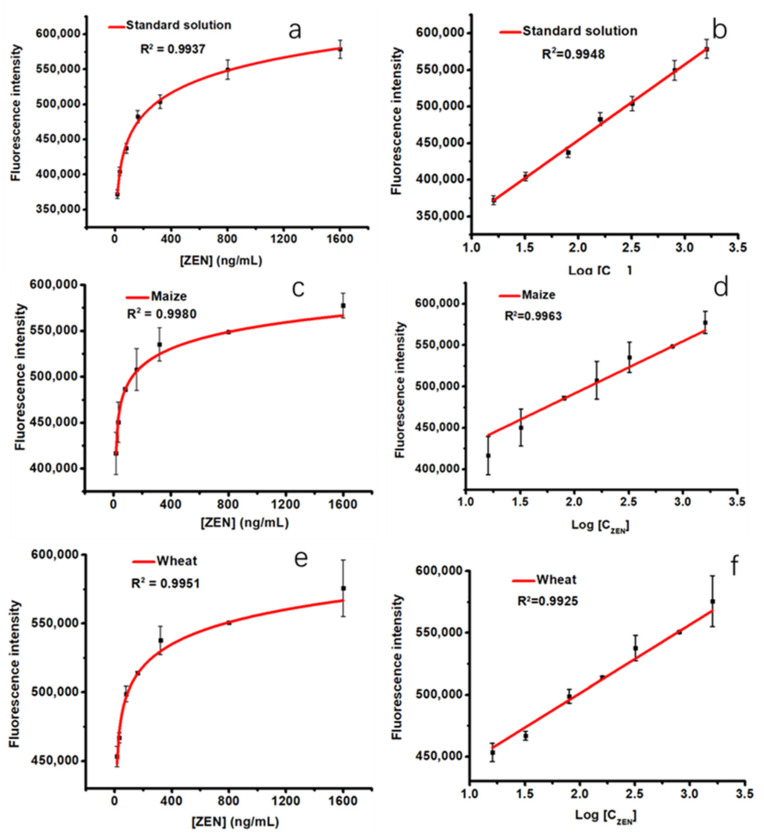
Curve fitting and calibration curves of the fluorescence intensity versus the various ZEN concentrations for the automated and high-throughput method ((**a**,**b**), spiked ZEN in standard solution; (**c**,**d**), spiked ZEN in maize matrix extraction, and (**e**,**f**), spiked ZEN in wheat matrix extraction).

To establish the calibration curve of ZEN using the automated magneto-controlled pretreatment and signal conversion system, a series of spiked samples of ZEN (16, 32, 80, 160, 320, 800, and 1600 ng/mL) were prepared. The fitting curve with a correlation coefficient of 0.9937 was obtained by fitting the ZEN concentration versus the corresponding fluorescence intensity (Figure 4a). The logarithmic value of ZEN concentration in the range of 16–1600 μg/kg was proportional to the fluorescence intensity (Y = 103,385.6x + 247,338.69, R^2^ = 0.9948) (Figure 4b). The limit of detection (LOD) was 0.33 μg/kg (S/N = 3), and the limit of quantification (LOQ) was 1.1 μg/kg (S/N = 10) based on the value of standard deviation (11,409.2) obtained from 0 ng/mL ZEN. In the analysis of the sample extracts (wheat and maize), the correlation coefficients were 0.9980 and 0.9951 for the curve fitting, respectively (Figure 4c,e). Good linear relationships (Y = 63,175.2x + 365,315.04, R^2^ = 0.9963 for wheat, and Y = 55,420.2x + 390,423.15, R^2^ = 0.9925 for maize) were also obtained between the fluorescence intensity and the logarithmic value of ZEN concentration (16–1600 μg/kg, Figure 4d,f). Relevant analytical parameters are presented in Table 1, indicating that our approach is effective for ZEN analysis in cereal samples. The analysis performance of this method is comparable to or even better than those of other ZEN detection assays [11,38,39,40,41,42,43,44] (Table S4).

To evaluate the recovery and repeatability for the CuO NP-based method, blank maize and wheat samples spiking at three different concentrations (30, 60, and 120 ng/mL) of ZEN were prepared. Through experiments and data analysis, Table 2 shows that the recovery rates were 99.2–104.9% and the intra-day relative standard deviation (RSD) were 0.7–5.1% for the two matrices, indicating that the recovery and precision of the automated and high-throughput method are satisfactory for ZEN detection in common cereals.

### 3.7. Specificity Analysis

The specificity is a significant parameter for achieving good performance of the developed method. To assess the specificity of the constructed assay, common mycotoxins including aflatoxin B_1_ (AFB_1_), ochratoxin (OTA), deoxynivalenol (DON), aflatoxin B_2_ (AFB_2_), aflatoxin G_1_ (AFG_1_), and aflatoxin G_2_ (AFG_2_), and a blank control were investigated. It can be seen in Figure S4 that only ZEN contributed to Cu^2+^ concentration due to the three-component competitive immune reaction of CuO NP-anti-ZEN, MB-BSA-ZEN, and ZEN. In contrast, high concentrations of Cu^2+^ were obtained when ZEN was replaced by other mycotoxins, and there was no apparent difference between them and the blank control, indicating that the designed assay can selectively detect ZEN in the presence of other mycotoxins.

### 3.8. Analytical Application for Actual Samples

**Figure 5:**
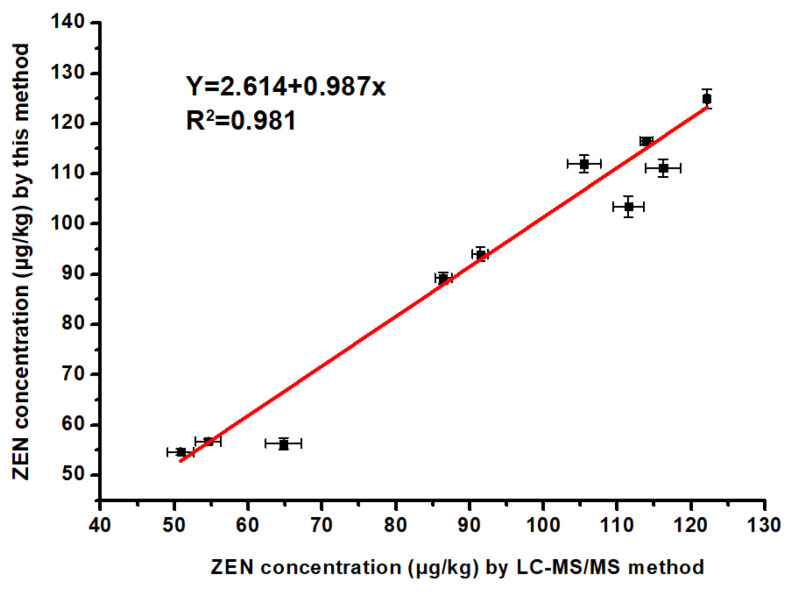
Comparison of determination results of 10 naturally contaminated samples analyzed through the CuO NP-based method and LC-MS/MS (*n* = 3).

The analytical reliability of the CuO NP-based assay for ZEN detection was evaluated by detecting 10 naturally contaminated samples. The performance of the constructed method was thoroughly verified by LC-MS/MS analysis. Figure 5 shows the regression analysis (Y = 2.614 + 0.987x, R^2^ = 0.981) between the two methods, demonstrating that the developed method agreed well with those obtained from LC-MS/MS. Thus, the automated and high-throughput approach was appropriate for determination samples with ZEN contamination.

## 4. Conclusions

A CuO NP-based immunosensor for ZEN detection was developed using an automated sample pretreatment and signal conversion system. This method takes advantage of three features of CuO NPs: effective adsorption of large amounts of antibodies as the capture probe; the ability to act as the signal source and signal carrier for signal amplification; and combined automated sample pre-processing with high-throughput terminal detection. Excellent accuracy, high recovery yield, and a wide linear range were achieved using this method. The developed assay simplifies tedious sample handling through automated processing and enables high-throughput terminal detection, which can facilitate the processing and inspection of massive samples. In addition, the assay can easily be extended to detect other mycotoxins.

## Figures and Tables

**Table 1:** The linearity, correlation coefficient, LOD, and LOQ for ZEN using the automated magneto-controlled pretreatment and signal conversion system in standard solution, maize, and wheat samples.

Matrix	Linearity Range (ng/mL)	Correlation Coefficient	LOD (ng/mL)	LOQ (ng/mL)
Standard solution	16–1600	0.9937	0.33	1.1
Maize	16–1600	0.9980	0.51	1.70
Wheat	16–1600	0.9951	0.61	2.02

**Table 2:** Recovery and relative standard deviations of spiked maize and wheat samples with various ZEN concentrations of three levels.

Sample	Added (ng/mL)	Detected (ng/mL)	Recovery (%, *n* = 3)	RSD (%, *n* = 3)
Maize	30	30.03	100.9	0.8
	60	59.76	99.6	5.1
	120	125.88	104.9	3.4
Wheat	30	29.88	99.6	0.7
	60	59.94	99.9	3.6
	120	119.04	99.2	3.7

## Data Availability

Not Applicable.

## Supplementary Materials

The following are available online at https://www.mdpi.com/article/10.3390/s21196538/s1, Figure S1: The structure of SGHK-Dns and the interaction principle between the SGHK-Dns and Cu^2+^. Figure S2: The study of fluorescence stability of SGHK-Dns (2 μM) with different concentrations of Cu^2+^ (0, 20, 50, 100, 200, 500, 1000 ng/mL), Figure S3: The logarithmic value of Cu^2+^ concentration in the range of 0–1000 ng/mL was proportional to the fluorescence intensity of SGHK-Dns (2 μM), Figure S4: The fluorescence intensity of the developed assay toward ZEN, AFB1, OTA, DON, AFB2, AFG1, AFG2, and blank control. The concentrations of AFB1, AFB2, AFG1, AFG2 were 20 ng/mL, the concentration of OTA, DON and ZEN were 20 ng/mL, 1000 ng/mL and 60 ng/mL, respectively, Table S1: the sequence of events for the automated magneto-controlled pretreatment and signal conversion process, Table S2: The fluorescence intensity when spiking ZEN (0, 16, 32, 80, 160, 320, 800, 1600 ng/mL) in different sample matrixes (PBS, Maize and Wheat), Table S3: Comparison of the analytical performances of our method with other ZEN detection assays, Table S4: An abbreviation list to the manuscript.
