# Supplementary material for: Copper Oxide Nanoparticle-Based Immunosensor for Zearalenone Analysis by Combining Automated Sample Pre-Processing and High-Throughput Terminal Detection
Source: Sensors (Basel). 2021 Sep 30;21(19):6538. doi: 10.3390/s21196538 (PMC8512712; doi:10.3390/s21196538)
Supplement: Supplementary file 1 [file sensors-21-06538-s001.zip › sensors-1346832-supplementary.pdf]

sensors

Copper Oxide Nanoparticle-Based Immunosensor for Zearalenone Analysis by Combining Automated Sample Pre-Processing and High-Throughput Terminal Detection

Zhihong Xuan, Yanxiang Wu, Hongmei Liu, Li Li, Jin Ye * and Songxue Wang *

Academy of National Food and Strategic Reserves Administration, No.11 Baiwanzhuang Str, Xicheng District, Beijing 100037, China; xzh@ags.ac.cn (Z.X.); wyx@ags.ac.cn (Y.W.); lhm@ags.ac.cn (H.L.); ll@ags.ac.cn (L.L.)
* Correspondence: yj@ags.ac.cn (J.Y.); wsx@ags.ac.cn (S.W.); Tel.: +86-010-5645-2662 (J.Y.); +86-010-5645-2668 (S.W.)

Citation: Xuan, Z.; Wu, Y.; Liu, H.; Li, L.; Ye, J.; Wang, S. Copper Oxide Nanoparticle-Based Immunosensor for Zearalenone Analysis by Combining Automated Sample Pre-Processing and High-Throughput Terminal Detection. *Sensors* **2021**, *21*, 6538. https://doi.org/10.3390/s21196538

Academic Editor: Sara Tombelli

Received: 3 August 2021
Accepted: 27 September 2021
Published: 30 September 2021

Publisher's Note: MDPI stays neutral with regard to jurisdictional claims in published maps and institutional affiliations.

Copyright: © 2021 by the authors. Licensee MDPI, Basel, Switzerland. This article is an open access article distributed under the terms and conditions of the Creative Commons Attribution (CC BY) license (http://creativecommons.org/licenses/by/4.0/).

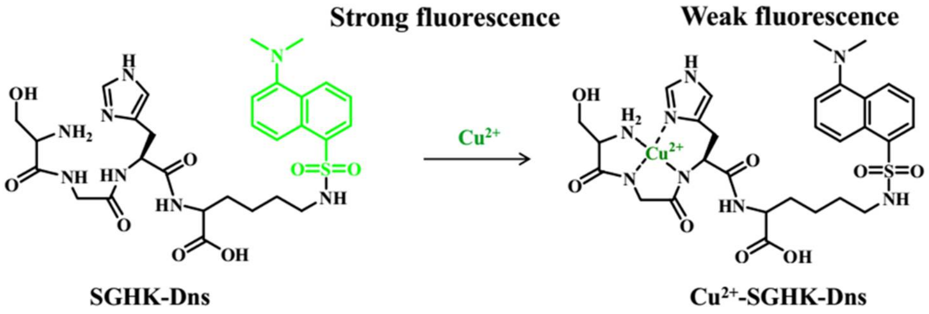

Figure S1. The structure of SGHK-Dns and the interaction principle between the SGHK-Dns and Cu^{2+}.

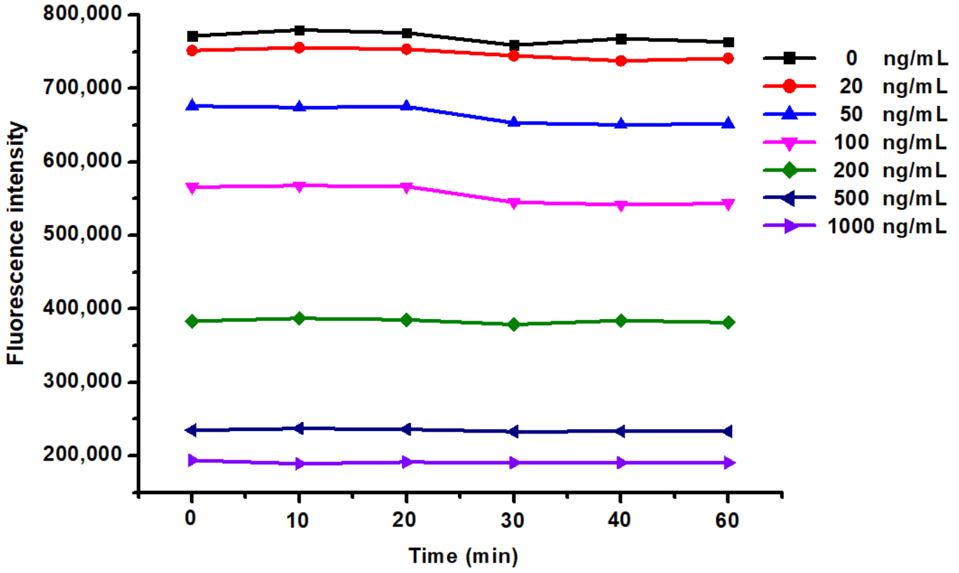

Figure S2. The study of fluorescence stability of SGHK-Dns (2μM) with different concentrations of Cu^{2+} (0, 20, 50, 100, 200, 500, 1000 ng/mL).

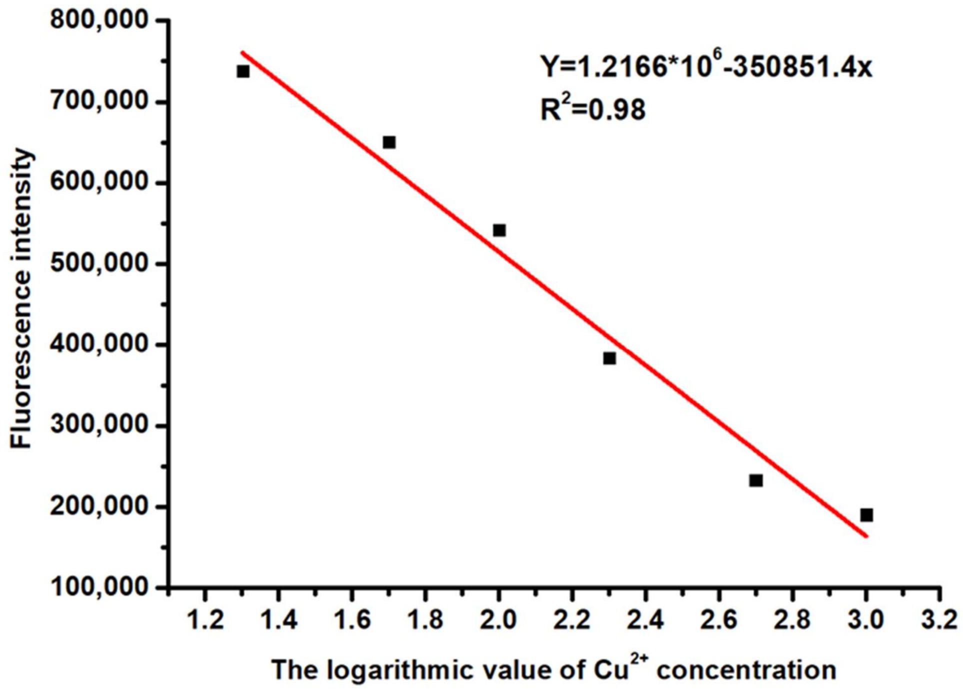

Figure S3. The logarithmic value of Cu^{2+} concentration in the range of 0-1000 ng/mL was proportional to the fluorescence intensity of SGHK-Dns (2μM).

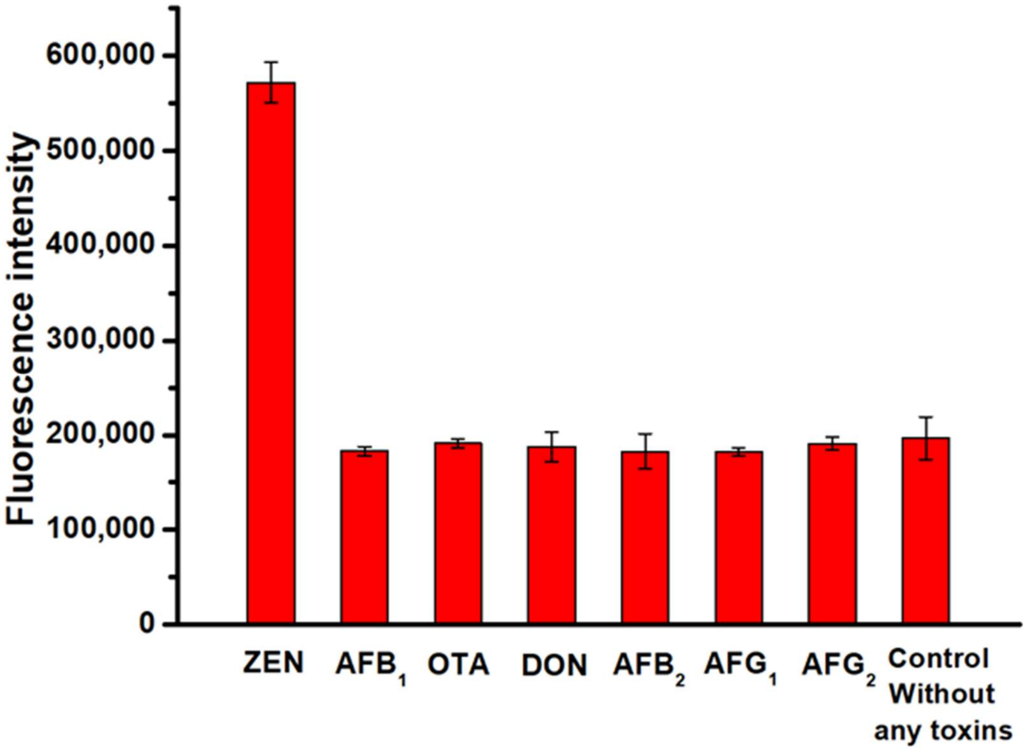

Figure S4. The fluorescence intensity of the developed assay toward ZEN, AFB_1, OTA, DON, AFB_2, AFG_1, AFG_2, and blank control. The concentrations of AFB_1, AFB_2, AFG_1, AFG_2 were 20 ng/mL, the concentration of OTA, DON and ZEN were 20 ng/mL, 1000 ng/mL and 60 ng/mL, respectively.

Table S1. The sequence of events for the automated magneto-controlled pretreatment and signal conversion process.

No.	Procedure	Mixing Time (min)	Volume (mL)
1	Competitive immune response (ZEN, CuO NP-anti-ZEN, MB-ZEN-BSA)	30.0	1.0
2	Washing with PBST	1.0	1.0
3	Washing with PBS	1.0	1.0
4	CuO NP Digestion	1.0	1.0
5	MB recycling	1.0	1.0

Table S2. The fluorescence intensity when spiking ZEN (0, 16, 32, 80, 160, 320, 800, 1600 ng/mL) in different sample matrixes (PBS, Maize and Wheat).

ZEN concentration (ng/mL)	Fluorescent Intensity				RSD
	PBS	Wheat	Maize	Average	
0	372,270	416,322	385,796	391,462.7	5.8%
16	396,421	453,353	416,600	422,124.7	6.8%
32	404,405	466,973	450,534	440,637.3	7.4%
80	437,365.5	498,878.5	486,084.5	474,109.5	6.8%
160	482,639.5	513,945.5	507,750	501,445	3.3%
320	504,064.5	537,702	535,267.5	525,678	3.6%
800	549,461.5	550,703	548,676.5	549,613.7	0.2%
1600	578,598.5	575,698	577,587.5	577,294.7	0.3%

Table S3. Comparison of the analytical performances of our method with other ZEN detection assays.

Target	Method	Materials	Detection Limit	Linear Range	Application of the Samples	Automated Processing	High Throughput Detection	Ref.
ZEN	Colorimetric	AuNPs	10 ng/mL	10–250 ng/mL	spiked corn and corn oil	No	No	[39]
ZEN	Fluorescence	Graphene oxide	0.5 ng/mL	1–16 ng/mL	spiked alcoholic, beverage samples, beer and wine	No	No	[40]
ZEN	Fluorescence	ssDNA aptamers	$7.85×10^{-10}$ M	$3.14×10^{-9}$ – $3.14×10^{-5}$ M	spiked beer samples	No	No	[41]
ZEN	Fluorescence	QDs	0.003–3.12 μM	0.002 μM	spiked corn, rice and wheat flours	No	No	[42]
ZEN	Fluorescence	QDs	0.6 or 1.5 ng/mL	–	spiked cereal samples	No	No	[43]
ZEN	Fluorescence	QDs	50 ng/mL	–	naturally contaminated wheat and maize samples	No	No	[44]
ZEN	Luminescece	C18 silica gel	–	–	cereals feedstuff	Yes	No	[45]
ZEN	Fluorescence	Carbon Quantum Dots	20 ng/mL	20–100 mg/L	spkied raw corn samples	No	No	[46]
ZEN	Fluorescence	CuNPs	0.33 ng/mL	16–1600 ng/mL	naturally contaminated wheat and maize samples	Yes	Yes	This method

Table S4. An abbreviation list to the manuscript.

ZEN	Zearalenone	BSA	Bovine serum albumin
CuO NPs	Copper oxide nanoparticles	NHS	N-hydroxysuccinimide
HPLC	High-performance liquid chromatography	MES	Morpholinoethanesulfonic acid
LC-MS/MS	Liquid chromatography combined with mass spectrometry	EDC	1-ethyl-3-(3-dimethylaminopropyl) carbodiimide hydrochloride
LFA	Lateral flow immunoassays	LOD	Limit of detection
AuNPs	Gold nanoparticles	LOQ	Limit of quantitation
ELISA	Enzyme-linked immunosorbent assays	SD	Standard deviation
MB	Magnetic bead	RSD	Relative standard deviation
QD	Quantum dot	AFB1	Aflatoxin B1
SERS	Surface-enhanced Raman scattering	AFB2	Aflatoxin B2
MOFs	Metal-organic frameworks	AFG1	Aflatoxin G1
DON	Deoxynivalenol	AFG2	Aflatoxin G2
IgG	Immunoglobulin G	OTA	OchratoxinA
Anti-ZEN	Monoclonal ZEN antibody	DLS	dynamic light scattering
